# Comparing del Nido and St. Thomas II cardioplegia in a rat ischemia–reperfusion model: Histopathology, mitochondria, and TEM analysis

**DOI:** 10.17305/bb.2025.13394

**Published:** 2025-12-23

**Authors:** Burak Toprak, Abdulkadir Bilgiç, Murat Özeren, Ebru Ballı

**Affiliations:** 1 Department of Cardiovascular Surgery, Mersin City Education and Research Hospital, Mersin, Türkiye; 2 Department of Cardiovascular Surgery, Mersin University Faculty of Medicine Hospital, Mersin, Türkiye; 3 Department of Histology, Mersin University Faculty of Medicine Hospital, Mersin, Türkiye

**Keywords:** Cardioplegia, myocardial ischemia, reperfusion injury, del Nido, St. Thomas II, transmission electron microscopy, histopathology, rat model

## Abstract

Myocardial ischemia–reperfusion (IR) injury remains a major challenge in cardiac surgery, and comparative histological and ultrastructural data on cardioplegia solutions are limited. This study compared the myocardial protective effects of St. Thomas II and del Nido cardioplegia in a controlled rat IR model, focusing on inflammation, mast cell dynamics, and subcellular preservation. Twenty-four Wistar Albino rats were randomized to Control, St. Thomas II, or del Nido groups. After 90 min of ischemia and 30 min of passive reperfusion, myocardial tissue was analyzed by hematoxylin–eosin, toluidine blue, and transmission electron microscopy (TEM). Outcomes included mast cell counts, leukocyte infiltration, karyolysis, and ultrastructural measures (Flameng score, crista density, basement membrane thickness). Both cardioplegia groups preserved myocardial morphology and attenuated inflammatory changes vs control. Light microscopy revealed a consistent mast cell density and reduced karyolysis in hearts treated with cardioplegia, with no significant differences observed between St. Thomas II and del Nido solutions. Conversely, TEM, the primary endpoint of this study, demonstrated enhanced mitochondrial and endothelial preservation in the del Nido group, as evidenced by significantly lower Flameng scores and increased crista density compared to both St. Thomas II and control groups (*P* < 0.05). In conclusion, both solutions reduced early IR-related injury, but del Nido provided a significant ultrastructural advantage on TEM despite similar routine light-microscopic findings.

## Introduction

Myocardial protection is a critical concern in cardiac surgery due to the ischemic period that follows aortic cross-clamping and cardiopulmonary bypass (CPB) [1, 2]. Ischemia–reperfusion (IR) injury significantly contributes to postoperative morbidity and mortality [3], prompting the development of various strategies—including hypothermia and cardioplegia—to mitigate this damage [4, 5].

Cardioplegia solutions suppress myocardial metabolism and reduce reperfusion injury by inducing diastolic arrest. However, the optimal composition and delivery methods of these solutions remain a topic of debate [6]. Among these, St. Thomas II is a classical crystalloid solution widely used in adults, providing reliable myocardial arrest but necessitating repeated dosing [7, 8]. In contrast, del Nido cardioplegia, originally designed for pediatric cases, has gained acceptance in adult populations due to its single-dose efficacy, sodium-channel blockade via lidocaine, and antioxidant properties from mannitol [9, 10]. St. Thomas II, a classical depolarizing crystalloid cardioplegia, contains high concentrations of potassium and magnesium, inducing diastolic arrest through membrane depolarization, while requiring repeated doses during prolonged ischemia. In contrast, del Nido cardioplegia is a polarized, low-calcium solution that offers extended single-dose arrest, augmented myocardial protection through sodium-channel blockade, and mannitol-mediated free-radical scavenging. These compositional and mechanistic differences form the conceptual basis for our comparison [7–10] and align with previous histological investigations of crystalloid cardioplegic formulations [8, 11]. Although some studies indicate comparable clinical outcomes between the two solutions regarding mortality and arrhythmias [11], few have explored their differential histological effects.

Mast cells, recognized for their role in early myocardial inflammation, contribute to IR injury through the release of histamine, cytokines, and proteases [12, 13]. However, their histological dynamics in cardioplegia-protected myocardium remain poorly characterized. Given their early activation and potential to amplify endothelial dysfunction, leukocyte infiltration, and tissue remodeling, mast cells represent a pivotal yet underexplored target for evaluating cardioprotective efficacy. In addition to conventional histological assessments, transmission electron microscopy (TEM) offers unique advantages in identifying ultrastructural alterations that precede visible light-microscopic injury. Mitochondrial swelling, cristae disruption, and sarcomeric disorganization are well-established early markers of IR damage [14]. Nevertheless, only a limited number of experimental studies have compared the ultrastructural impacts of cardioplegic solutions using TEM, with recent investigations by Jung et al. providing preliminary evidence for solution-dependent mitochondrial preservation [15]. Incorporating TEM allows for a more sensitive evaluation of myocardial preservation at the subcellular level, complementing traditional staining methods and enhancing the histopathological interpretation of cardioplegic efficacy. Therefore, this study aimed to compare the histopathological efficacy of St. Thomas II and del Nido cardioplegia in a rat IR model, focusing on mast cell activity and myocardial structural preservation.

## Materials and methods

### Study design

This study was conducted as a randomized controlled animal experiment at the Departments of Cardiovascular Surgery and Histology-Embryology, Faculty of Medicine, Mersin University. The objective was to compare the histopathological effects of St. Thomas II and del Nido cardioplegia solutions on myocardial ischemia and reperfusion injury. A total of 24 male young adult Wistar Albino rats (aged 3 months) were utilized. The rats were randomly assigned into three groups, each subdivided into ischemia and reperfusion subgroups: Group 1 (Control group), Group 2 (St. Thomas II cardioplegia group), and Group 3 (del Nido cardioplegia group). Randomization codes were generated before the experimental procedures and recorded as anonymous group labels. The observer conducting histopathological and TEM analyses had access only to these anonymized codes, ensuring blinding to the actual treatment allocation. Group 1 was designated as the Control (IR without cardioplegia) since these animals underwent the same IR phase sequence but received saline instead of cardioplegia.

### Data collection

#### Animal housing and acclimatization

All animals were housed in standard polypropylene cages (2–3 per cage) under a 12-h light/dark cycle, with ambient temperatures maintained at 22–24 ^∘^C and humidity at 50%–60%. They had ad libitum access to food and water, with environmental enrichment provided through paper nesting and polyvinyl chloride (PVC) tubes. All rats were acclimatized for at least seven days before experimentation.

#### Anesthesia and surgical preparation

Rats were anesthetized using intraperitoneal ketamine (75 mg/kg) and xylazine (10 mg/kg). Adequate anesthesia was confirmed through pedal withdrawal and corneal reflex tests. Endotracheal intubation was not necessary; spontaneous respiration was maintained during thoracotomy, and after the chest was opened, ventilation was managed via passive open-chest mechanics, a well-established method in non-survival IR models. A midline sternotomy was performed, and the ascending aorta was cannulated using standard microsurgical techniques. Following the administration of cardioplegia or saline, the myocardium entered complete chemical arrest, with spontaneous respiration gradually ceasing as expected in a non-perfused setting. Local tissue trauma was minimized, and no additional analgesia was necessary since all procedures were non-survival. Euthanasia was performed using an intraperitoneal overdose of pentobarbital (200 mg/kg) in accordance with American Veterinary Medical Association (AVMA) recommendations. All procedures complied with Animal Research: Reporting of *In Vivo* Experiments (ARRIVE) guidelines and institutional animal-welfare regulations.

#### Autologous blood collection for the del Nido group

Autologous blood (1 mL) was collected only in the del Nido group via a brief midline laparotomy and inferior vena cava puncture to prepare the blood–crystalloid formulation. No abdominal procedures were performed in the St. Thomas II or control groups. This blood-collection step is inherent to the preparation of blood-containing del Nido cardioplegia and is widely employed in experimental cardioplegia models; therefore, its use in only one study arm represents a methodological requirement rather than a procedural imbalance.

Direct access to the aortic root or atrial structures without prior sternotomy is technically unfeasible in rats. In contrast, the inferior vena cava can be safely accessed via a brief laparotomy without compromising thoracic structures, allowing rapid, atraumatic blood collection under stable hemodynamic conditions. Hemostasis was achieved before proceeding with the protocol.

Since all subsequent steps—including ischemia, reperfusion, fixation, and histological/TEM analyses—were identical, this additional blood-collection step was unlikely to influence myocardial ultrastructure in a non-survival model.

#### Preparation and administration of cardioplegia

The del Nido solution was prepared by combining 1 part autologous blood with 4 parts Plasma-Lyte A, resulting in a final hematocrit of approximately 6%–8%. The ionic composition included Na^+^ 128–132 mEq/L, K^+^ 26 mEq/L, Mg^2+^ 16 mEq/L, Ca^2+^ <1.0 mEq/L, bicarbonate, mannitol, and lidocaine (Table 1). The working pH was maintained at 7.35–7.45 at a temperature of 22–24 ^∘^C.

**Table 1:** Composition of prepared cardioplegia (per 1000 mL)

**Component**	**St. Thomas II**	**del Nido**
Base Solution	Ringer’s Lactate 1000 mL	Plasma-Lyte A 1000 mL
Sodium (Na^+^)	110 mEq	–
Magnesium (Mg^2+^)	32 mEq	16 mEq
Potassium (K^+^)	16 mEq	26 mEq
Calcium (Ca^2+^)	2.4 mEq	–
Sodium Bicarbonate (8.4%)	10 mL	13 mL
Mannitol (20%)	–	16.3 mL
Lidocaine (1%)	–	13 mL
pH	7.8	7.4

This table displays the component concentrations of St. Thomas II and del Nido cardioplegia solutions per 1000 mL. No statistical tests were conducted, as this is a compositional comparison. Concentrations are expressed in milliequivalents (mEq) or milliliters (mL), and pH is presented as a unitless value.

### Administration volumes

- **del Nido:** single 5 mL dose via the aortic root
- **St. Thomas II:** four 4 mL doses at 20-minute intervals
- **Control:** 4 mL of 0.9% saline

All solutions were infused at 4–6 ^∘^C. To ensure procedural consistency, saline in the control group was administered through the same aortic cannula.

#### Ischemia protocol and temperature control

Cardiac arrest was confirmed visually by the absence of atrial pulsation and complete cessation of ventricular movement. Continuous electrocardiography (ECG) monitoring was not utilized, in accordance with non-survival IR protocols. A fixed ischemic duration of 90 min was imposed on all subjects. Repeated doses of St. Thomas II were administered within this timeframe without interrupting total ischemia. Myocardial temperature was maintained at 30–32 ^∘^C using sterile crushed ice packs positioned near (but not in direct contact with) the heart. Surface temperature was continuously monitored with a calibrated thermometer, while systemic normothermia was supported using a heating platform.

#### Passive reperfusion protocol (Table 2)

At the conclusion of the ischemic period, the heart was excised and submerged in normothermic (36.5 ^∘^C) isotonic saline for a 30-minute passive reperfusion period (Table 2).

**Table 2:** Experimental protocol: Passive reperfusion phase

**Time segment**	**Group 1 (Control)**	**Group 2 (St. Thomas II)**	**Group 3 (del Nido)**
0–5 min	Anesthesia and surgical preparation	Anesthesia and surgical preparation	Anesthesia and surgical preparation
5–6 min	0.9% saline administration (placebo)	First dose of St. Thomas II cardioplegia	Single dose of del Nido cardioplegia
6–96 min	Ischemia (total 90 minutes)	Ischemia (total 90 minutes) with repeated doses at approx. 26, 47, 68 min	Ischemia (total 90 minutes)
26 min	–	Second St. Thomas II dose (within 90-min ischemia)	–
47 min	–	Third St. Thomas II dose (within 90-min ischemia)	–
68 min	–	Fourth St. Thomas II dose (within 90-min ischemia)	–
96–126 min	Passive reperfusion (normothermic saline immersion)	Passive reperfusion (normothermic saline immersion)	Passive reperfusion (normothermic saline immersion)
126–129 min	Tissue sampling (reperfusion group)	Tissue sampling (reperfusion group)	Tissue sampling (reperfusion group)

This schematic table presents a comprehensive summary of the experimental protocol for three groups: Control, St. Thomas II, and del Nido. The timeline encompasses anesthesia and surgical preparation, cardioplegia administration (when applicable), a fixed 90-minute ischemic period, and a subsequent 30-minute reperfusion phase. Tissue samples were collected at specified time points for both ischemia and reperfusion groups. No statistical analyses were conducted on this procedural flow. It is important to note that the 30-minute post-ischemic phase involves passive normothermic saline immersion rather than genuine hemodynamic reperfusion.

**Table 3:** Composition of the reperfusion solution utilized across all groups

**Component**	**Concentration**
Sodium chloride (NaCl)	0.9% (w/v)
Potassium (K^+^)	3.0 mmol/L
Calcium (Ca^2+^)	1.2 mmol/L
pH (at 36.5^∘^C)	7.35–7.40
Temperature	36.5^∘^C

The reperfusion solution comprised modified isotonic saline supplemented with K^+^ 3.0 mmol/L and Ca^2+^ 1.2 mmol/L to prevent contracture and calcium paradox during rewarming (Table 3). Due to the absence of oxygen, glucose, or perfusate flow, recovery was achieved exclusively through passive diffusion rather than hemodynamic reperfusion. This early-phase IR model facilitates controlled rewarming and cardioplegia washout without the confounding variables of shear forces or pressure. Consequently, all reperfusion findings in this study pertain to passive *ex vivo* reperfusion. This streamlined approach, adapted from Jung et al., allows for gradual metabolic reactivation and cardioplegia washout without mechanical perfusion [15].

#### Tissue sampling for light microscopy (LM) and TEM

Following the 30-min reperfusion period, the left ventricle was divided into two equal transverse sections:

**Basal half** → fixed in 10% neutral-buffered formalin (NBF) for LM

**Apical half** → immersed in 2.5% glutaraldehyde for TEM

Sampling was performed within **60–90 s** to minimize autolytic changes. For TEM, specimens were post-fixed in osmium tetroxide, dehydrated through graded alcohols, transitioned with propylene oxide, and embedded in epoxy resin prior to ultrathin sectioning.

#### Histopathological analysis

Mast cell counts were conducted manually across 10 non-overlapping fields at 200× magnification per specimen, including both granulated and degranulated mast cells. Leukocyte infiltration was assessed in five 400× fields and scored from 0 to 3, yielding a cumulative infiltration score of 0–15. Nuclear integrity (karyolysis) was graded from 0 to 3 at 400× magnification. All analyses were performed by a blinded histologist.

#### Transmission electron microscopy

Ultrathin sections (70–80 nm) were prepared and analyzed using a JEOL JEM-1400 Plus system at 120 kV, equipped with a calibrated OneView 4K complementary metal–oxide–semiconductor (CMOS) camera. A minimum of ≥10 non-overlapping fields were sampled from each animal. The primary endpoint for TEM was the Flameng mitochondrial injury score (0–4), with ≥100 mitochondria evaluated per rat; inferential statistics utilized animal-level summary values.

Secondary endpoints included:

- Cristae density
- Mitochondrial morphometry (area, perimeter, aspect ratio, form factor)
- Z-line integrity
- Sarcolemmal blebbing
- Capillary ultrastructure

#### Sample size and exclusion criteria

Power analysis indicated that 6–7 rats per group were adequate; thus, 8 rats per group were utilized (*n* ═ 24).

**Inclusion criteria:** Male Wistar Albino rats, aged 3 months, weighing 200–300 g.

**Exclusion criteria:** Failed cannulation, intraoperative mortality, instability, or inadequate tissue quality. No animals met the exclusion criteria.

### Ethical statement

This study received ethical approval from the Mersin University Animal Experiments Ethics Committee (Approval No: 2023/28, Date: 10.01.2023).

### Statistical analysis

Quantitative data from histopathological evaluations were analyzed using SPSS 24.0 statistical software (IBM Corporation, Armonk, NY, USA). Normality of TEM-derived continuous variables was assessed using the Shapiro–Wilk test. Between-group comparisons were conducted using the Kruskal–Wallis test with Dunn–Bonferroni post-hoc tests; effect sizes were reported as η^2^ (Kruskal–Wallis) or *r* (pairwise). Given the conservative nature of the Bonferroni-adjusted post-hoc tests and the limited number of primary endpoints, no additional false-discovery-rate (FDR) corrections were applied. For non-parametric rank-based tests, effect size estimates (η^2^ and *r*) were provided; however, 95% CIs were not reported, as standardized CI computation for these non-Gaussian effect size metrics is not methodologically established. Categorical and ordinal scores (e.g., Flameng and Z-line integrity) were analyzed similarly using non-parametric methods, reported as median (interquartile range). Inter-rater agreement for primary and secondary endpoints was quantified using intraclass correlation coefficient (ICC) (2,1) with 95% CIs, and agreement was visualized with Bland–Altman plots. A two-sided *P*-value < 0.05 was deemed statistically significant. The normality of data distribution was further evaluated using the Kolmogorov–Smirnov test. As LM scores were ordinal, intergroup comparisons were performed with the Kruskal–Wallis test followed by Dunn–Bonferroni post-hoc analysis where appropriate. Within each treatment group, paired comparisons between ischemia and reperfusion phases were conducted using the Wilcoxon signed-rank test, accounting for repeated measurements from the same animals. For non-normally distributed data, the Kruskal–Wallis test was applied, and where significant, pairwise comparisons were completed with the Dunn–Bonferroni correction. All continuous and ordinal variables are presented as median (interquartile range), aligning with the non-parametric analytical framework. A two-tailed *P*-value < 0.05 was considered statistically significant across all analyses. Histopathological parameters such as macrophage count and inflammation scores were evaluated by a blinded observer for each tissue sample, thereby minimizing observer bias. All histopathological endpoints derived from multiple microscopic fields (mast cell counts, leukocyte infiltration scores, and karyolysis) were aggregated at the specimen or animal level (sum or mean across fields), and only these aggregated animal-level values were included in group-wise statistical comparisons to prevent pseudoreplication. Results were additionally visualized with box plots. No sensitivity re-analysis was conducted beyond the primary non-parametric comparisons, as the small sample size and ordinal endpoints precluded additional modeling.

## Results

A total of 24 Wistar Albino rats, each weighing between 200–300 g, were included in the study. The rats were randomly assigned to three groups: Group 1 (Control group – physiological saline, *n* ═ 8), Group 2 (St. Thomas II cardioplegia group, *n* ═ 8), and Group 3 (del Nido cardioplegia group, *n* ═ 8). Tissue samples were collected during both the ischemia and reperfusion phases for all groups.

**Figure 1.**
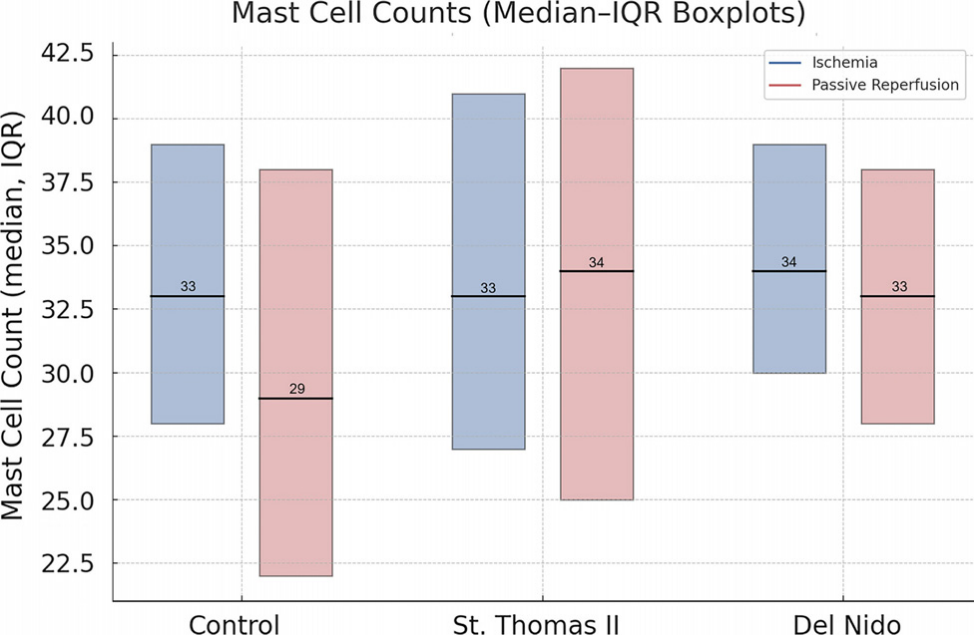
**Distribution of mast cell counts during ischemia and reperfusion among the Control, St. Thomas II, and del Nido groups.** Data are presented as median and interquartile range, visualized using boxplots. The Control group exhibits a significant decrease in mast cell numbers from ischemia to reperfusion (33 (28–39) → 29 (22–38), *P* ═ 0.02). In contrast, no significant phase-based changes were observed in either the St. Thomas II or del Nido groups.

Mast cell counts were manually assessed in toluidine blue-stained sections at 200× magnification, examining 10 distinct fields per specimen. The results are summarized in Table 4. During the ischemia phase, mast cell counts were comparable among the groups, with median values of 33 (28–39) in the control group, 33 (27–41) in the St. Thomas II group, and 34 (30–39) in the del Nido group, indicating no significant intergroup differences (*P* ═ 0.95). In the reperfusion phase, median mast cell counts were 29 (22–38), 34 (25–42), and 33 (28–38), respectively, again showing no statistically significant differences between groups (*P* ═ 0.55). Within-group analysis revealed a significant reduction in mast cell count from ischemia to reperfusion only in the control group (*P* ═ 0.02), while the St. Thomas II (*P* ═ 0.19) and del Nido (*P* ═ 0.61) groups showed no significant changes between phases (Table 4). This finding is illustrated in Figure 1.

**Table 4:** Comparison of mast cell counts across groups and phases

**Phase**	**Control (*n* ═ 8)**	**St. Thomas II (*n* ═ 8)**	**del Nido (*n* ═ 8)**	***P* value**
Ischemia	33 (28–39)	33 (27–41)	34 (30–39)	0.95
Reperfusion	29 (22–38)	34 (25–42)	33 (28–38)	0.55
***P* value**	**0.02**	0.19	0.61	–

Light microscopic ordinal scores, including mast cell counts, leukocytic infiltration, and karyolysis, were analyzed utilizing nonparametric tests. Between-group comparisons were conducted using Kruskal–Wallis tests with Dunn–Bonferroni post-hoc corrections. Data are presented as median (interquartile range, IQR). In the table, statistically significant values are indicated in bold. The *P* value denotes the level of statistical significance, with values less than 0.05 deemed significant.

Leukocytic infiltration scores were evaluated in hematoxylin–eosin-stained sections at 400× magnification by counting extravascular leukocytes in five randomly selected fields per sample. The leukocytic infiltration scores, which ranged from 0 to 15, were derived from the sum of five high-power fields. The scoring system was as follows: 0 (none), 1 (<20 leukocytes), 2 (20–45 leukocytes), and 3 (>45 leukocytes). The scores are detailed in Table 5.

**Table 5:** Comparative analysis of leukocytic infiltration scores by group and phase

**Phase**	**Control (*n* ═ 8)**	**St. Thomas II (*n* ═ 8)**	**del Nido (*n* ═ 8)**	***P* value**
Ischemia	9 (8–10)	10 (10–11)	9 (7–11)	0.10
Reperfusion	10 (9–10)	10 (10–11)	10 (9–10)	0.15
***P* value**	0.11	0.99	0.49	–

Light microscopic ordinal scores, including mast cell counts, leukocytic infiltration, and karyolysis, were analyzed using nonparametric tests. Between-group comparisons were conducted with Kruskal–Wallis tests followed by Dunn–Bonferroni post-hoc corrections. Data are presented as medians with interquartile ranges (IQR). In the table, statistically significant values are highlighted in bold. The *P* value reflects the level of statistical significance, with values less than 0.05 deemed significant.

**Figure 2.**
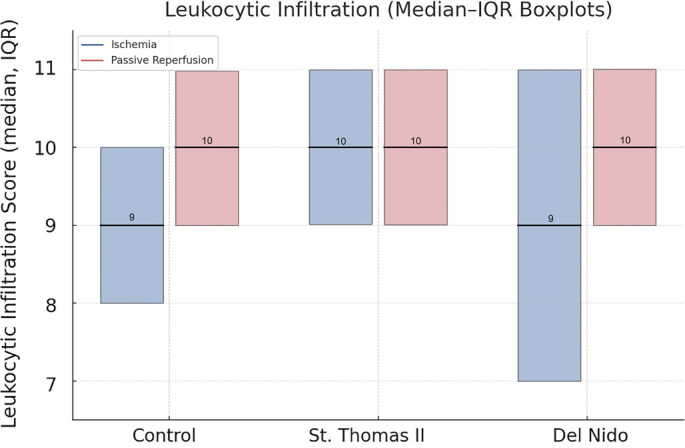
**Comparison of leukocytic infiltration scores in ischemia (blue) and reperfusion (red) phases across control, St. Thomas II, and del Nido groups.** Data are presented as median and interquartile ranges using boxplot visualization. No statistically significant change was observed within or between groups (*P* > 0.05), indicating that cardioplegia application did not significantly alter leukocyte infiltration.

During the ischemia phase, leukocytic infiltration scores were similar across groups, with median values of 9 (8–10) in the control group, 10 (10–11) in the St. Thomas II group, and 9 (7–11) in the del Nido group (*P* ═ 0.10). In the reperfusion phase, scores were 10 (9–10), 10 (10–11), and 10 (9–10), respectively, indicating no significant intergroup differences (*P* ═ 0.15). Within-group evaluations demonstrated that leukocytic infiltration did not significantly change from ischemia to reperfusion in the control (*P* ═ 0.11), St. Thomas II (*P* ═ 0.99), or del Nido (*P* ═ 0.49) groups (Table 5), as illustrated in Figure 2.

In addition to mast cell counts and leukocytic infiltration, nuclear integrity was semi-quantitatively assessed by scoring karyolytic nuclei within cardiac myocytes in H&E-stained sections. Karyolysis, characterized by faded or absent nuclear staining, was evaluated in five random fields per section under 400× magnification. A scoring system ranging from 0 (no karyolysis) to 3 (widespread karyolysis) was utilized. For each specimen, the scores from these fields were averaged to produce a single animal-level karyolysis score per phase for statistical analysis. The scores are presented in Table 6. Karyolytic nuclear scores showed similar distributions across groups during the ischemia phase, with median values of 2 (1–2) for the control, St. Thomas II, and del Nido groups. During reperfusion, the control group exhibited an increase to 2 (2–3), which was statistically significant compared to its ischemia value (*P* ═ 0.03). Conversely, both cardioplegia groups maintained stable karyolysis scores between ischemia and reperfusion (St. Thomas II: *P* ═ 0.12; del Nido: *P* ═ 0.31), indicating preservation of nuclear morphology under cardioplegic protection. No significant between-group differences were observed in either phase (Table 6).

**Table 6:** Semi-quantitative assessment of karyolytic nuclei in myocardial tissue during ischemia and reperfusion phases

**Group**	**Ischemia phase (median, IQR)**	**Reperfusion phase (median, IQR)**	***P* value (within group)**
Control	2 (1–2)	2 (2–3)	0.03*
St. Thomas II	2 (1–2)	2 (1–2)	0.12
del Nido	2 (1–2)	2 (1–2)	0.31

Karyolytic nuclear scores during the ischemia and reperfusion phases were evaluated across experimental groups. Karyolysis was graded on a scale of 0 to 3 in H&E-stained sections. A statistically significant increase in karyolytic changes was observed in the control group following reperfusion (*P* ═ 0.03), whereas the cardioplegia-treated groups exhibited more stable nuclear morphology. Light microscopic ordinal scores, including mast cell counts, leukocyte infiltration, and karyolysis, were analyzed using nonparametric tests. Between-group comparisons were conducted using Kruskal–Wallis tests with Dunn–Bonferroni post-hoc correction. Data are presented as median (interquartile range, IQR).

**Figure 3.**
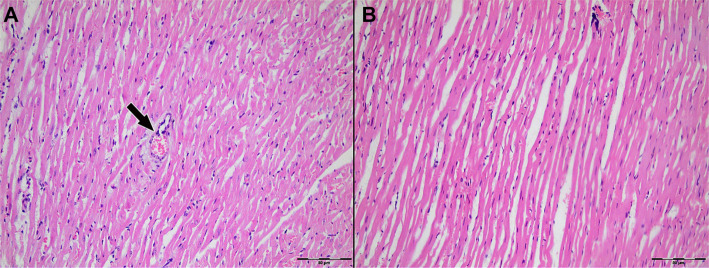
**Representative H&E-stained sections of left ventricular myocardium from Group 1 (Control; IR without cardioplegia).** (A) Ischemia phase; (B) Reperfusion phase. Cardiomyocytes show preserved overall morphology with regular fiber alignment, homogeneous cytoplasmic staining, and centrally located nuclei; interstitial microvascular profiles remain intact. The arrow indicates an intramyocardial blood vessel. Original magnification ×200. Abbreviations: H&E: Hematoxylin–eosin; IR: Ischemia–reperfusion.

**Figure 4.**
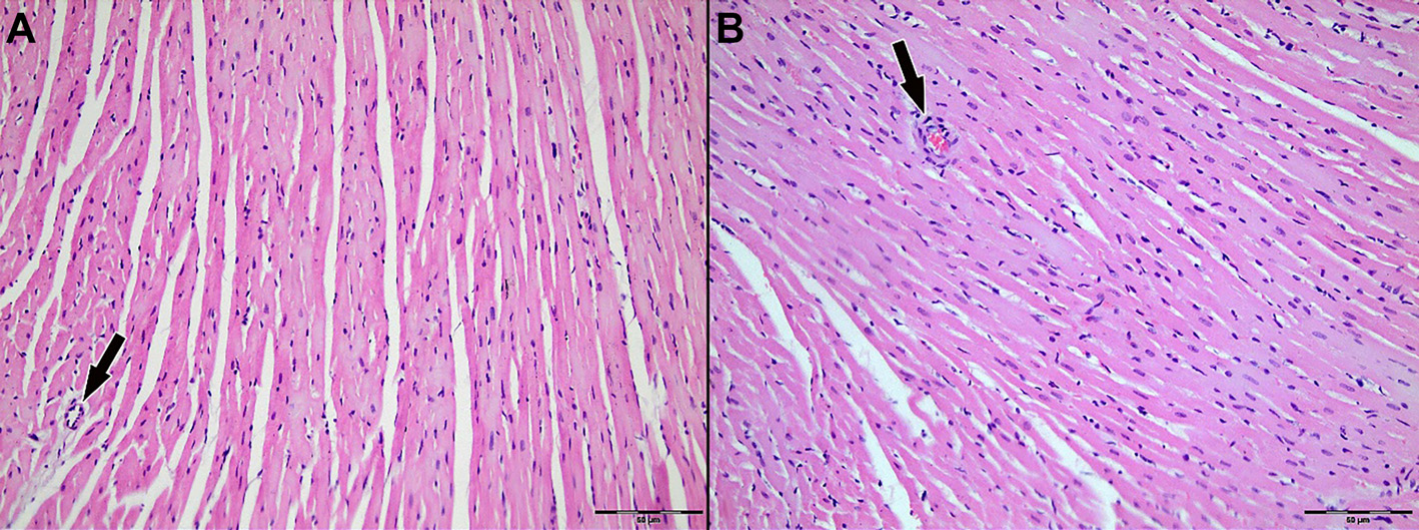
**Representative H&E-stained sections of left ventricular myocardium from Group 2 (St. Thomas II cardioplegia).** (A) Ischemia phase; (B) Reperfusion phase. Cardiomyocytes demonstrate preserved architecture with regular alignment, homogeneous cytoplasmic staining, and intact, centrally located nuclei; interstitial microvascular profiles remain morphologically intact. Arrows indicate intramyocardial blood vessels. Original magnification ×200. Abbreviation: H&E: Hematoxylin–eosin.

**Figure 5.**
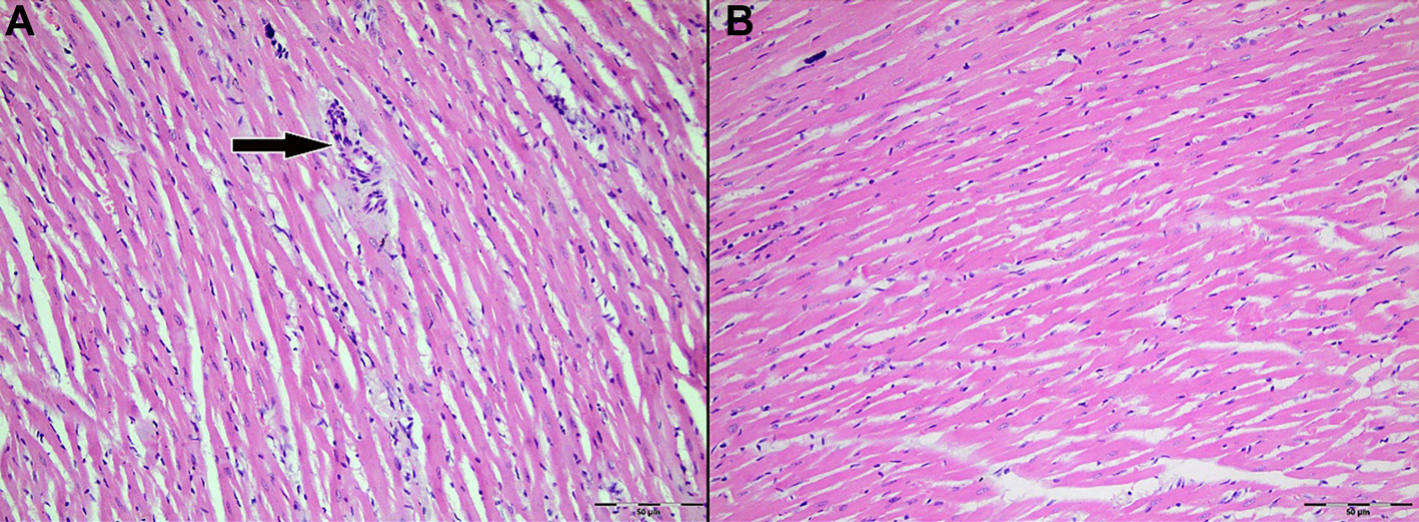
**Representative hematoxylin–eosin (H&E)-stained sections of left ventricular myocardium from Group 3 (del Nido cardioplegia).** (A) Ischemia phase; (B) Reperfusion phase. Cardiomyocytes show preserved overall morphology with regular fiber alignment, homogeneous cytoplasmic staining, and centrally located nuclei; interstitial microvascular profiles between myocytes remain intact. The arrow indicates an intramyocardial blood vessel. Original magnification ×200. Abbreviation: H&E: Hematoxylin–eosin.

Light microscopic examination of H&E-stained myocardial tissue sections from all groups revealed preserved general morphology of cardiac muscle cells. Cardiomyocytes exhibited regular alignment, homogeneously stained cytoplasm, and centrally located nuclei. Capillary structures between myocytes retained their normal anatomical appearance (Figures 3–5).

**Figure 6.**
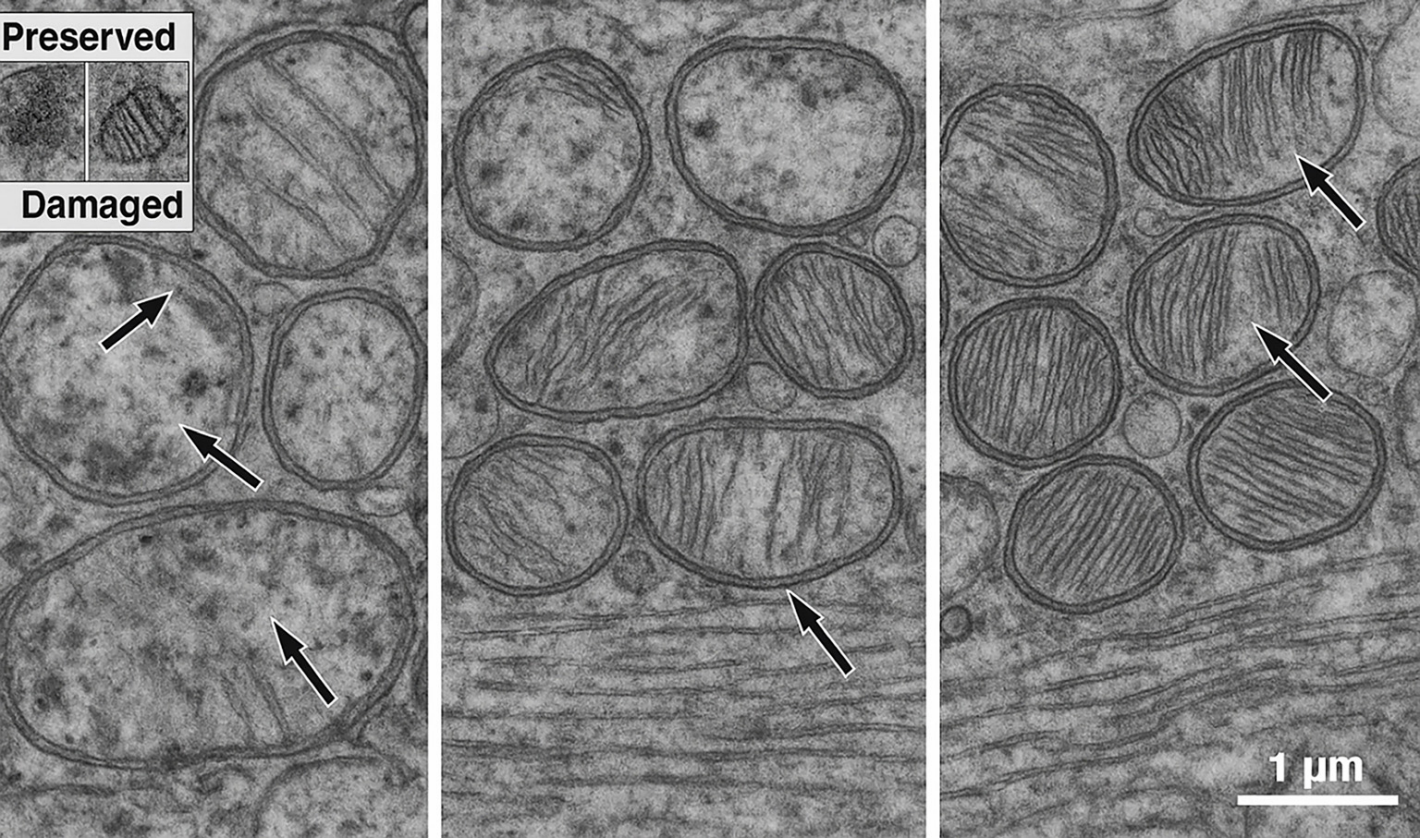
**Representative transmission electron micrographs depicting mitochondrial ultrastructure following ischemia–reperfusion injury.** The transmission electron microscopy images illustrate mitochondrial morphology across the experimental groups: Left, Control; middle, St. Thomas II; right, del Nido. In the Control panel (left), arrows indicate severely swollen mitochondria, disrupted and fragmented cristae, and areas of matrix rarefaction. In the St. Thomas II panel (middle), arrows highlight partially preserved cristae with moderate mitochondrial swelling. In the del Nido panel (right), arrows denote well-preserved cristae, dense matrices, and intact outer and inner membranes. An inset panel (upper left) provides a direct comparison of preserved vs damaged mitochondria, illustrating intact cristae in the preserved example and crista disruption in the damaged example. All micrographs were captured at approximately ×25,000 magnification, with scale bars representing 1 µm.

TEM evaluation of the Control group revealed extensive mitochondrial injury, characterized by cristae disruption, matrix clearing, and irregular outer membranes. St. Thomas II cardioplegia provided moderate ultrastructural preservation, although focal swelling and partial cristae rarefaction persisted. In contrast, myocardium treated with del Nido solution exhibited nearly intact mitochondrial morphology, characterized by dense, orderly cristae and minimal swelling. The median Flameng score was significantly lower in the del Nido group compared to both the Control and St. Thomas II groups (*P* < 0.05), confirming superior preservation of mitochondrial integrity (Figure 6).

Quantitative ultrastructural scoring revealed a progressive decline in mitochondrial injury across groups. The Control myocardium exhibited the highest Flameng scores (median = 3.0, IQR = 2.5–3.5), consistent with severe swelling and cristae disruption. The St. Thomas II group demonstrated intermediate scores (median = 2.0, IQR = 1.5–2.5), indicating partial mitochondrial preservation. The del Nido group achieved the lowest scores (median = 0.5, IQR = 0–1.0), reflecting well-preserved mitochondrial integrity and dense cristae.

**Figure 7.**
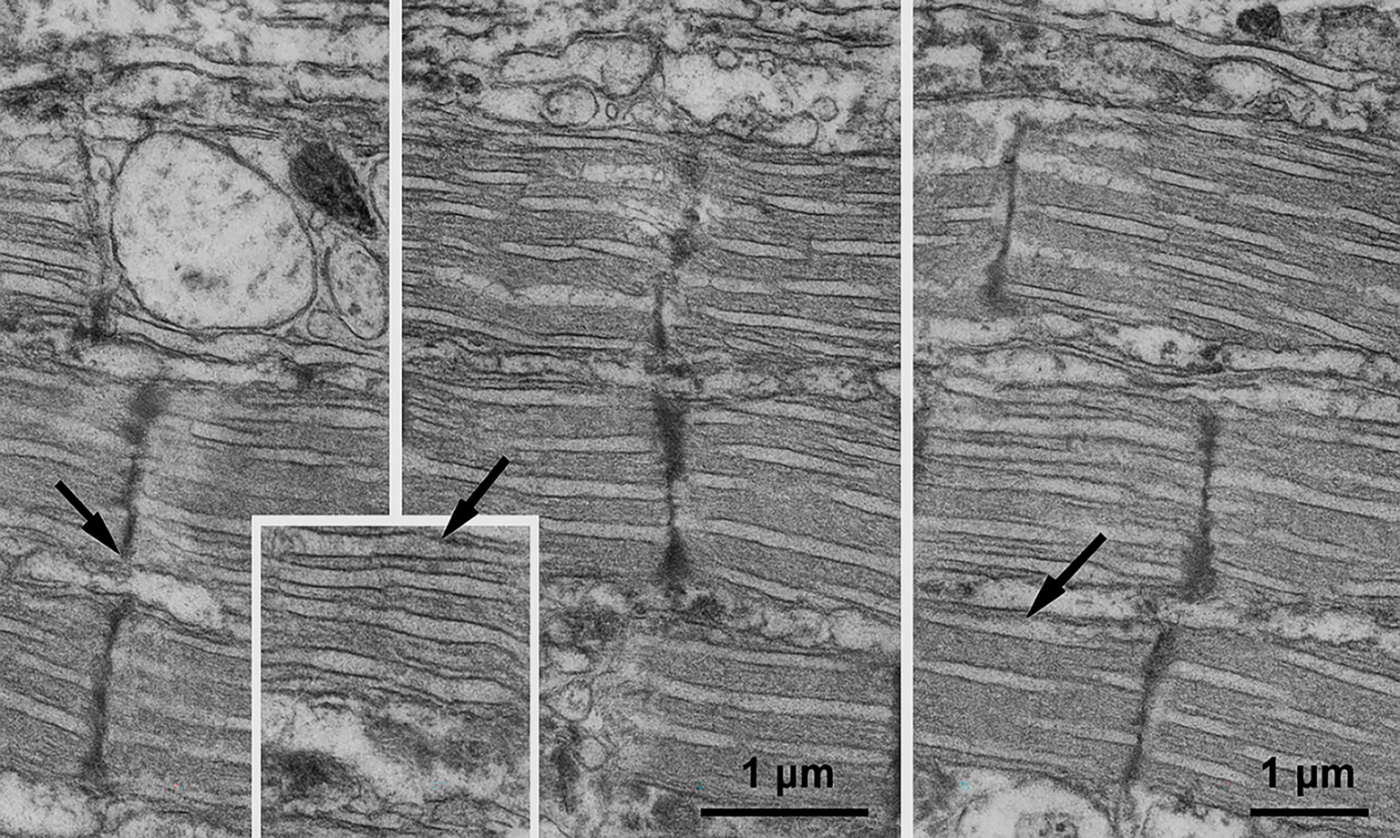
**Transmission electron micrographs depicting sarcomeric organization and sarcolemmal morphology following ischemia–reperfusion in rat myocardium.** The panels are organized as follows: Left, Control; middle, St. Thomas II; right, del Nido. Black arrows indicate areas of sarcolemmal irregularity or blebbing. The Control group exhibits disrupted sarcomeric alignment and focal discontinuities in the sarcolemma. The St. Thomas II group shows partially preserved banding patterns with mild irregularities. In contrast, the del Nido group presents a more uniform sarcomeric periodicity and smoother sarcolemmal contours. The inset panel offers a magnified view of a well-preserved sarcomeric region from the del Nido sample. All images were captured at approximately ×18,000 magnification, with scale bars representing 1 µm.

These differences were statistically significant (*P* < 0.05), aligning with the morphological observations in Figure 6 and supporting the superior protective efficacy of del Nido cardioplegia against IR-induced mitochondrial injury (Figure 7).

**Figure 8.**
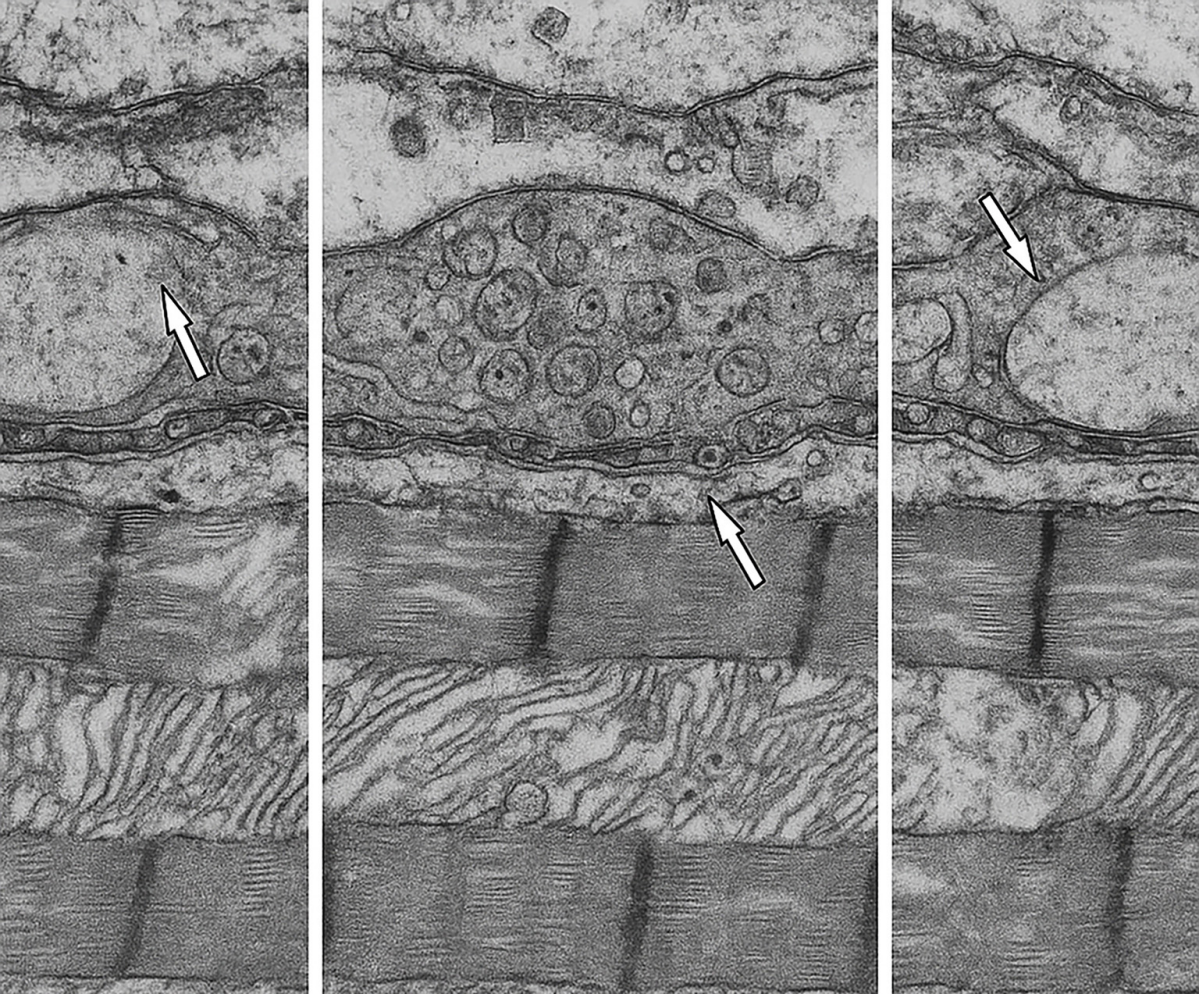
**Transmission electron micrographs of myocardial capillary and endothelial ultrastructure following ischemia–reperfusion.** Representative TEM images illustrate the ultrastructural changes in myocardial capillaries across the experimental groups: Left, Control; middle, St. Thomas II; right, del Nido. In the Control panel (left), arrows indicate severe endothelial swelling, abundant pinocytic vesicles, and markedly thickened basement membranes. In the St. Thomas II panel (middle), arrows highlight localized endothelial protrusions and vesicle clusters, demonstrating partial preservation with focal vesicular dilatation. In the del Nido panel (right), arrows point to thin basement membrane segments and a continuous endothelial lining, reflecting minimal swelling, preserved membrane integrity, and reduced vesiculation. All micrographs were obtained at approximately ×25,000 magnification, with scale bars representing 500 nm. Abbreviation: TEM: Transmission electron microscopy.

Quantitative ultrastructural assessment demonstrated significant improvements in both crista density and mitochondrial form factor in the del Nido group compared to the Control and St. Thomas II groups. Crista density values were 38.2 (30.5–46.1) intersections/µm^2^ in the Control group, 55.4 (49.8–60.9) in the St. Thomas II group, and 68.7 (63.1–72.5) in the del Nido group (*P* < 0.05). The progressive increase in mitochondrial form factor (0.72 → 0.86) reflects a more circular and structurally preserved mitochondrial morphology, indicating reduced outer-membrane distortion and improved crista organization. These results corroborate the ultrastructural observations and support the enhanced mitochondrial preservation afforded by del Nido cardioplegia (Figure 8).

Quantitative ultrastructural analysis indicated significant group differences across all measured parameters. The Flameng score was lowest in the del Nido group (median 0.5, IQR 0–1.0), indicating minimal mitochondrial damage, while the Control group exhibited the most severe injury (median 3.0, IQR 2.5–3.5; *P* < 0.001). Both crista density and form factor were significantly higher in the del Nido myocardium compared to the St. Thomas II and Control groups (*P* < 0.05), reflecting superior preservation of mitochondrial architecture. Additionally, Z-line continuity and basement membrane thickness favored the del Nido group, which exhibited the thinnest basement membranes and the most organized sarcomeric alignment. The overall effect sizes (η^2^ ═ 0.61–0.78) indicated a strong influence of cardioplegia type on ultrastructural preservation (Table 7).

**Table 7:** Quantitative ultrastructural parameters of myocardium observed via TEM

**Parameter**	**Control**	**St. Thomas II**	**del Nido**	***P* value**	**Effect size (η^2^/r)**
Flameng score	3.0 (2.5–3.5)	2.0 (1.5–2.5)	0.5 (0–1.0)	**<0.001**	0.78
Crista density (intersections/µm^2^)	38.2 (30.5–46.1)	55.4 (49.8–60.9)	68.7 (63.1–72.5)	**<0.001**	0.72
Form factor (4π ·area/perimeter^2^)	0.72 (0.68–0.76)	0.79 (0.75–0.82)	0.86 (0.84–0.88)	**0.002**	0.61
Z-line integrity score	2.8 (2.5–3.0)	1.9 (1.5–2.3)	0.7 (0.5–1.2)	**<0.001**	0.65
Basement membrane thickness (nm)	196 (178–215)	154 (140–165)	122 (113–134)	**<0.001**	0.70

Values are reported as median (IQR). Statistical analysis was conducted using the Kruskal–Wallis test, followed by Dunn–Bonferroni correction. A *P* value of less than 0.05 was considered statistically significant. Effect sizes are expressed as η^2^ for the Kruskal–Wallis test and as r for pairwise comparisons. Abbreviations: TEM: Transmission electron microscopy; IQR: Interquartile range.

Pairwise post-hoc analysis revealed that the del Nido group exhibited significantly lower Flameng scores and basement membrane thickness, as well as higher crista density and form factor values compared with both the Control and St. Thomas II groups (*P* < 0.05 for all). The St. Thomas II group also demonstrated partial protection compared to Control, particularly in crista density and Z-line continuity. Overall, del Nido cardioplegia provided the most effective ultrastructural preservation, reflected by the largest effect sizes (*r* ═ 0.62–0.84) across multiple morphological parameters. These findings align with qualitative TEM observations and quantitative violin/boxplot distributions, confirming a consistent protective pattern at the mitochondrial, sarcomeric, and endothelial levels (Table 8).

**Table 8:** Post-hoc Dunn–Bonferroni pairwise comparisons of quantitative TEM parameters

**Parameter**	**Control vs St. Thomas II**	**Control vs del Nido**	**St. Thomas II vs del Nido**
Flameng score (0–4)	***P* ═ 0.030**, r = 0.51	***P* < 0.001**, r = 0.83	***P* ═ 0.010**, r = 0.56
Crista density (intersections/µm^2^)	***P* < 0.001**, r = 0.78	***P* < 0.001**, r = 0.86	***P* ═ 0.040**, r = 0.45
Form factor (4π ·area/perimeter^2^)	*P* ═ 0.060, r = 0.36	***P* ═ 0.002**, r = 0.62	***P* ═ 0.030**, r = 0.48
Z-line integrity (0–3)	***P* ═ 0.002**, r = 0.64	***P* < 0.001**, r = 0.80	***P* ═ 0.010**, r = 0.55
Basement membrane thickness (nm)	***P* < 0.001**, r = 0.72	***P* < 0.001**, r = 0.84	***P* ═ 0.020**, r = 0.50

The Dunn–Bonferroni correction was implemented, with a two-tailed *P* value threshold of <0.05 deemed statistically significant. Effect sizes (r) were calculated based on pairwise nonparametric rank comparisons.

**Figure 9.**
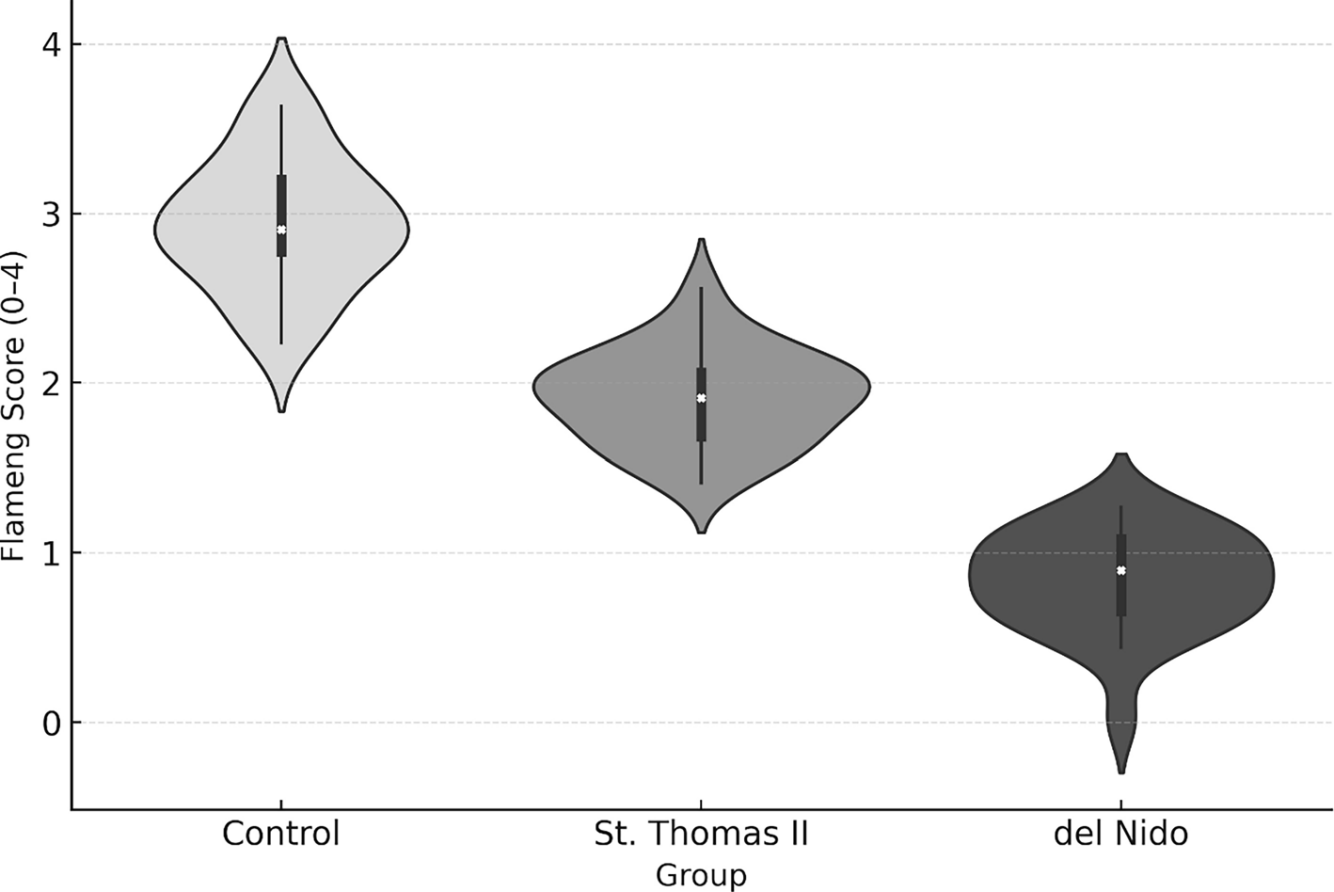
**Distribution of flameng scores across experimental groups.** Violin and box plots illustrate the distribution of mitochondrial Flameng injury scores among the study groups. The median scores were highest in the Control group, moderate in the St. Thomas II group, and lowest in the del Nido group. The broad distribution observed in the Control group indicates heterogeneous and severe mitochondrial injury, while the del Nido group exhibits a compact clustering around lower scores (0–1), signifying minimal ultrastructural damage. Statistical analysis using the Kruskal–Wallis test followed by Dunn–Bonferroni correction confirmed a significant reduction in Flameng scores in the del Nido group compared to both the Control and St. Thomas II groups (*P* < 0.05).

Ultrastructural evaluation revealed significant sarcomeric and sarcolemmal disruption in the Control group, including loss of Z-line continuity and cytoplasmic blebbing. The St. Thomas II group demonstrated partial structural preservation but still exhibited mild sarcomeric disorganization. In contrast, the del Nido group maintained uniform sarcomere alignment and clear A–I band differentiation with preserved sarcolemmal integrity. Quantitative scoring indicated that the del Nido group had significantly lower median sarcomeric disruption scores compared with the Control and St. Thomas II groups (*P* < 0.05), indicating enhanced myofibrillar protection (Figure 9).

**Figure 10.**
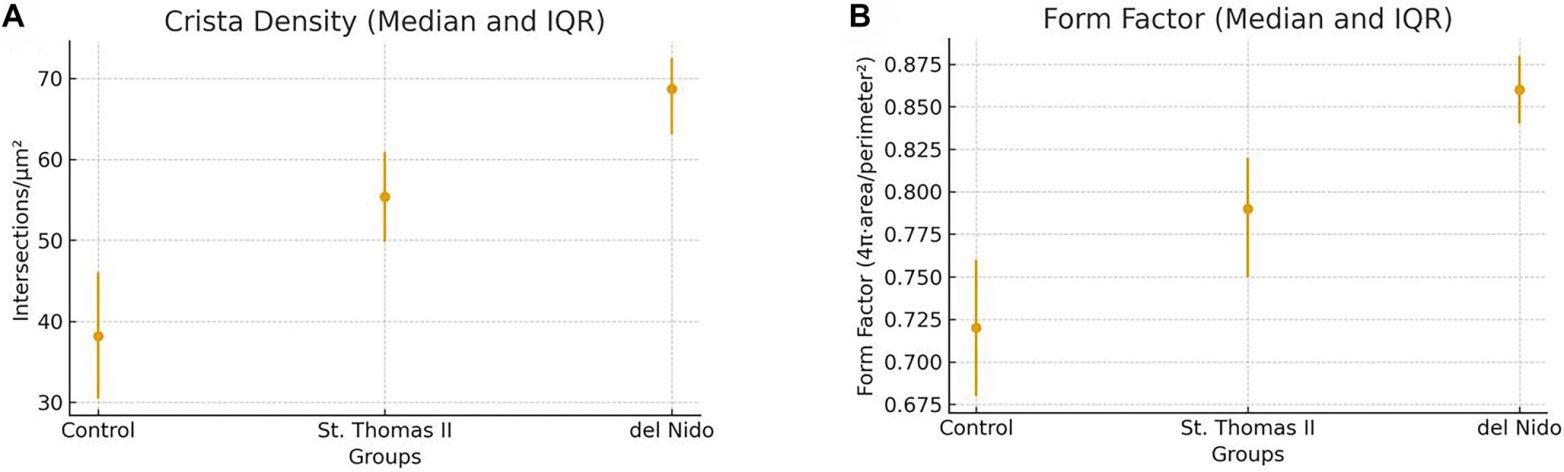
**Quantitative mitochondrial ultrastructural metrics across experimental groups (TEM morphometry).** (A) Crista density (intersections/µm^2^) presented as median with interquartile range (IQR). The Control group showed the lowest crista density, St. Thomas II demonstrated intermediate preservation, and the del Nido group exhibited the highest crista density, consistent with superior maintenance of crista organization. (B) Mitochondrial form factor (4π ·area/perimeter^2^) presented as median with IQR. Lower values in the Control group indicate greater contour irregularity and outer-membrane distortion; St. Thomas II showed intermediate preservation, whereas del Nido exhibited the most regular mitochondrial morphology. Error bars denote the IQR. Abbreviations: TEM: Transmission electron microscopy; IQR: Interquartile range.

Ultrastructural assessment revealed that IR caused severe endothelial injury in the Control group, characterized by cytoplasmic edema, vesicle accumulation, and basement membrane thickening. St. Thomas II cardioplegia provided partial preservation of endothelial morphology but did not completely prevent subendothelial edema. Conversely, the del Nido group maintained a more continuous endothelial lining with thinner basement membranes and visibly fewer pinocytic vesicles. These observations are presented qualitatively, as vesicle counts were not included among the quantitative TEM parameters in Tables 7 and 8. Collectively, these qualitative findings support an enhanced microvascular protective effect in the del Nido group (Figure 10A and B).

**Figure 11.**
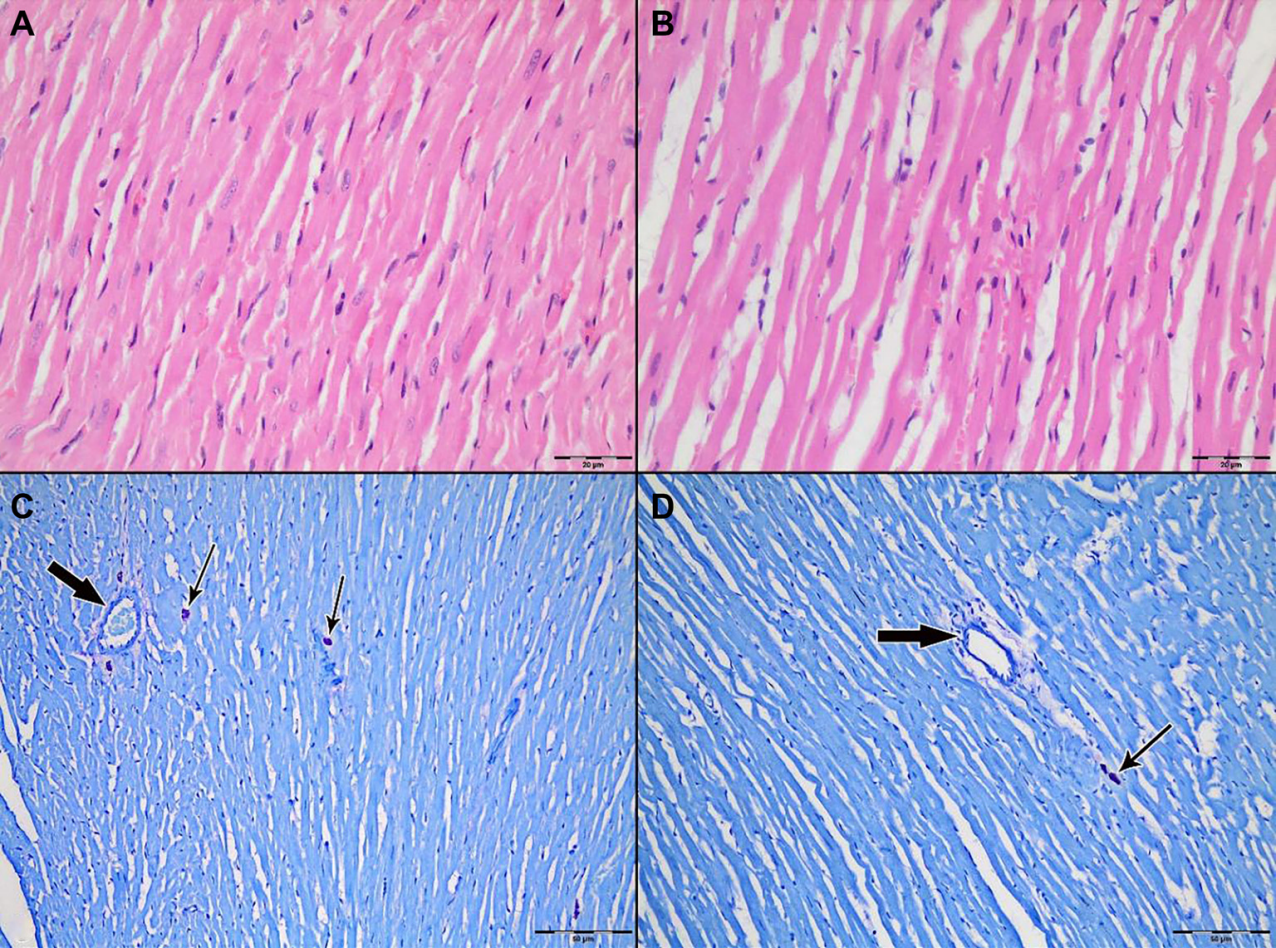
**Light microscopic evaluation of myocardium from Group 1 (Control; ischemia–reperfusion without cardioplegia).** (A and C) Ischemia phase; (B and D) reperfusion phase. (A and B) Representative hematoxylin–eosin (H&E) sections (original magnification ×400) illustrating overall myocardial architecture. (C and D) Toluidine blue–stained sections (original magnification ×200) highlighting mast cells (thin arrows) in perivascular/interstitial regions; intramyocardial blood vessels are indicated by thick arrows. A visible reduction in mast cell density is observed after reperfusion (D) compared with ischemia (C), consistent with quantitative analysis (33 [28–39] vs 29 [22–38]; *P* ═ 0.02).

**Figure 12.**
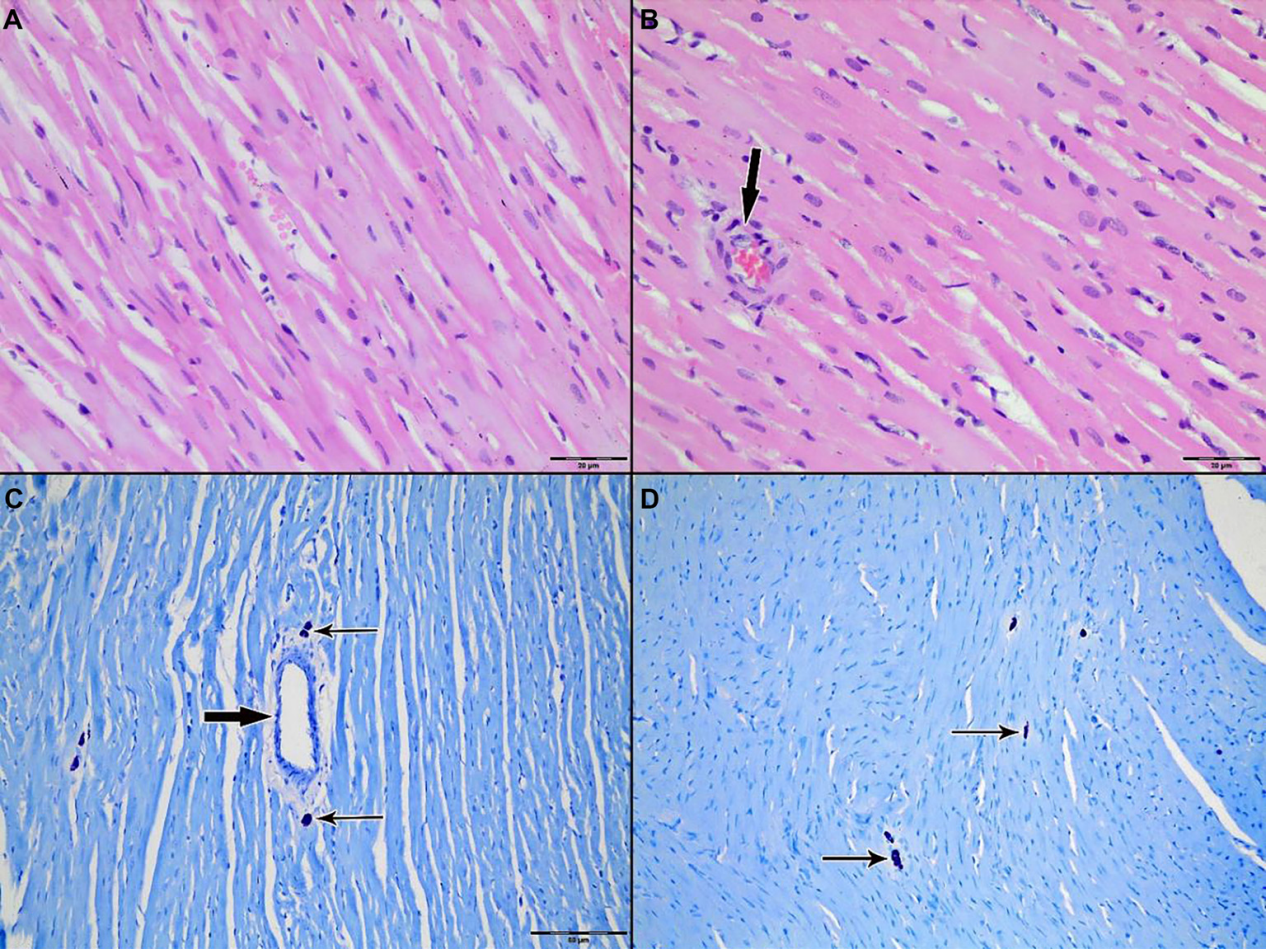
**Light microscopic evaluation of myocardium from Group 2 (St. Thomas II cardioplegia).** (A and C) Ischemia phase; (B and D) reperfusion phase. (A and B) Representative H&E sections (original magnification ×400) demonstrating preserved myocardial architecture. (C and D) Toluidine blue–stained sections (original magnification ×200) highlighting mast cells (thin arrows) in perivascular/interstitial regions; intramyocardial blood vessels are indicated by thick arrows. Mast cell density appears comparable between ischemia and reperfusion, consistent with quantitative counts (33 [27–41] vs 34 [25–42]; *P* ═ 0.19). Abbreviation: H&E: Hematoxylin–eosin.

**Figure 13.**
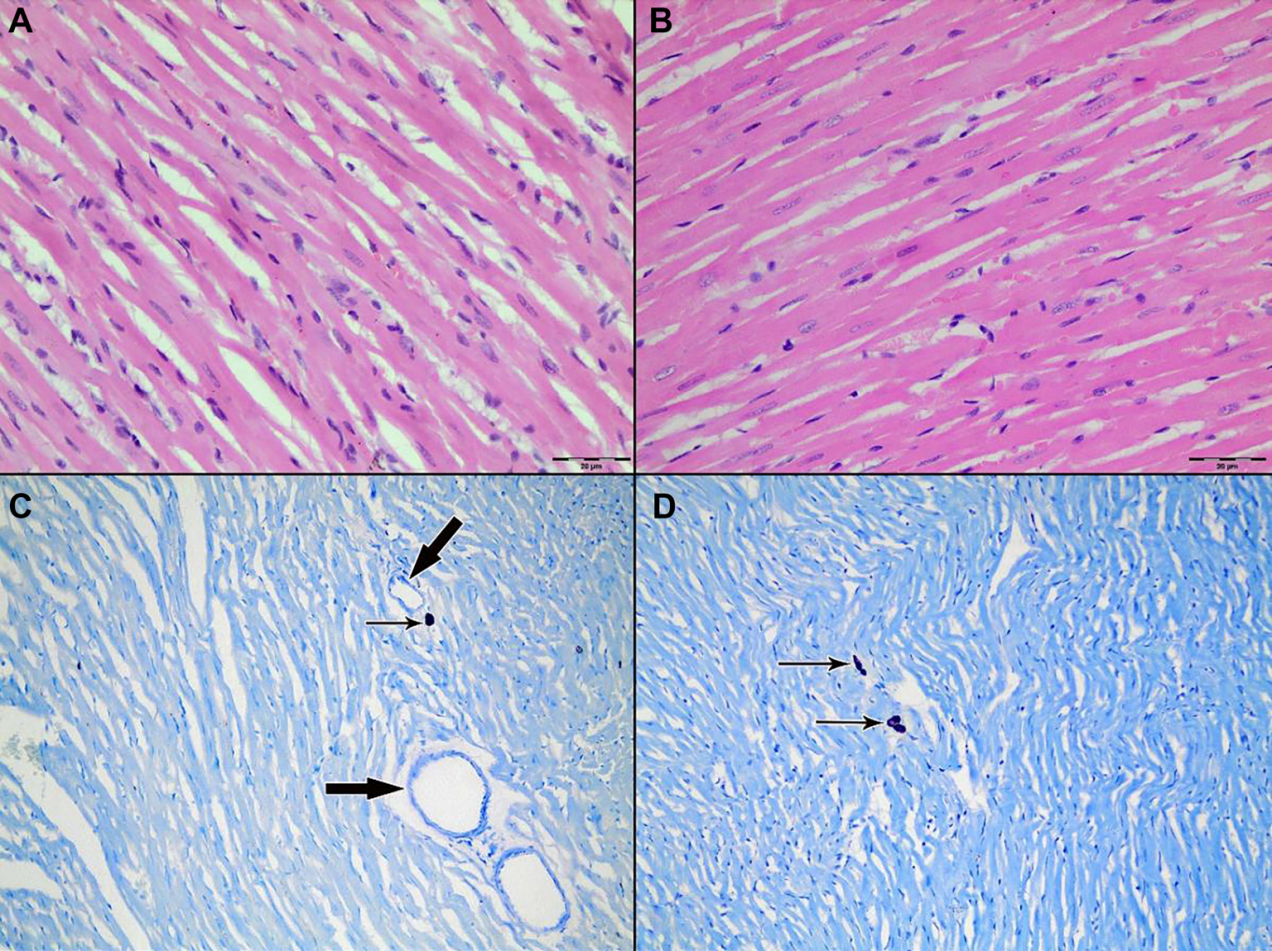
**Light microscopic evaluation of myocardium from Group 3 (del Nido cardioplegia).** (A and C) Ischemia phase; (B and D) reperfusion phase. (A and B) Representative H&E sections (original magnification ×400) showing preserved myocardial architecture. (C and D) Toluidine blue-stained sections (original magnification ×200) highlighting mast cells (thin arrows) in perivascular/interstitial regions; intramyocardial blood vessels are indicated by thick arrows. Mast cell density appears stable between ischemia and reperfusion, consistent with quantitative counts (34 [30–39] vs 33 [28–38]; *P* ═ 0.61). Abbreviation: H&E: Hematoxylin–eosin.

To assess the degree of inflammation, leukocytic infiltration scoring was conducted on H&E-stained sections. No statistically significant differences were observed among the three groups during either the ischemia or reperfusion phases. While leukocytic infiltration was predominantly perivascular, a mild increase in inflammatory cell numbers was noted in the control group after reperfusion; however, this increase was not statistically significant (Figures 11–13).

When comparing mast cell counts between the ischemia and reperfusion phases in the control group, a statistically significant decrease was observed during reperfusion (33 [28–39] → 29 [22–38], *P* ═ 0.02) (Table 4). In contrast, no significant changes were noted in the St. Thomas II group (33 [27–41] vs 34 [25–42], *P* ═ 0.19) or the del Nido group (34 [30–39] vs 33 [28–38], *P* ═ 0.61). Mast cells were particularly concentrated in the interstitial and perivascular areas. In toluidine blue-stained slides, mast cells exhibited intense purple staining and retained their characteristic granular structures (Figures 11–13).

Figure 11 illustrates a visible decrease in mast cell numbers in the control group following reperfusion. Quantitative analysis supports this finding, demonstrating a significant reduction from 33 (28–39) during ischemia to 29 (22–38) during reperfusion (*P* ═ 0.02). Figures 12 and 13 depict Groups 2 and 3, respectively, where mast cell counts remained stable during reperfusion. Specifically, in the St. Thomas II group, mast cell counts were 33 (27–41) during ischemia and 34 (25–42) during reperfusion (*P* ═ 0.19), while the del Nido group showed counts of 34 (30–39) vs 33 (28–38) (*P* ═ 0.61). These histological findings align with the quantitative data presented in Table 4. The decrease in mast cell numbers during reperfusion in the control group may indicate mast cell degranulation or early cell loss, both anticipated outcomes of unprotected IR injury. In contrast, the stable mast cell counts observed in both cardioplegia groups suggest that cardioplegia likely mitigated acute degranulation-related mast cell depletion. Figure legends denote mast cells (thin black arrows) and blood vessels (thick black arrows).

Additionally, macrophage counts, another cellular parameter assessed in this study, were not quantitatively evaluated due to the lack of specific immunohistochemical staining. However, light microscopic observations indicated increased cellular accumulation in the interstitial space, particularly in the control group after reperfusion. This accumulation likely included macrophages within the mononuclear cell population, suggesting a more active inflammatory cascade in tissues not exposed to cardioplegia.

In conclusion, microscopic examinations and histological staining results demonstrated that both cardioplegia solutions preserved myocardial morphological integrity and limited the inflammatory response to reperfusion to some extent. Collectively, these findings indicate that both cardioplegia solutions offer protective effects against IR injury and effectively stabilize mast cell responses. Although no statistically significant differences were identified between the St. Thomas II and del Nido groups, graphical and microscopic evaluations suggest that the del Nido group exhibited a more controlled inflammatory response. This observation may reflect the synergistic effects of its pharmacological components; however, confirmation would require extended reperfusion models and molecular assays. The significant increase in mast cell count noted in the control group implies that reperfusion injury is more pronounced in the absence of cardioplegia.

## Discussion

Myocardial protection during surgery is crucial for reducing both intraoperative and postoperative complications. In open-heart surgery, cardioplegia not only facilitates temporary cardiac arrest but also plays a critical role in preserving organ function in the postoperative period. By inducing cardiac arrest, metabolic demands on the heart are reduced, and oxygen consumption is minimized [16]. The selection of an appropriate cardioplegia solution is vital for maintaining myocardial integrity throughout the surgical procedure [8]. Currently, both crystalloid and blood-based cardioplegia solutions are utilized, with parameters such as ion composition, pH, and viscosity directly influencing their effects on myocardial tissue [17, 18].

St. Thomas II, a classical crystalloid cardioplegia solution widely used in adult cardiac surgery, is rich in potassium and magnesium. It induces diastolic arrest through cell membrane depolarization and reduces circadian energy expenditure [19]. Conversely, del Nido cardioplegia, initially developed for pediatric surgery, has proven effective in adult cases due to its lower dosing requirement and longer duration of action [20]. A distinguishing feature of del Nido is its lidocaine content, which blocks sodium channels and reduces intracellular calcium accumulation, along with mannitol, which acts as a free radical scavenger [21]. This mechanism limits myocyte injury related to calcium overload, particularly during the reperfusion phase, emphasizing del Nido’s pharmacological advantages [8]. Despite its theoretical suitability for mitigating reperfusion injury, our study did not demonstrate significant histopathological superiority.

In our study employing a rat model of experimental myocardial ischemia and reperfusion, we compared the light microscopic (histopathological) effects of St. Thomas II and del Nido cardioplegia solutions. Light microscopic evaluations revealed no significant differences in leukocytic infiltration scores between groups; however, the overall inflammatory response appeared less pronounced in the cardioplegia-treated groups, particularly in the del Nido group. Although this trend did not reach statistical significance, it may reflect qualitative histological benefits associated with cardioplegic protection. In toluidine blue-stained sections, mast cell counts were significantly lower in the control group after reperfusion, decreasing from 33 (28–39) to 29 (22–38), suggesting a more intense inflammatory response in the absence of cardioplegia. These findings imply that mast cell loss due to reperfusion occurred primarily in the control group, while both cardioplegia-treated groups demonstrated relative mast cell stability, indicating a potential protective role of cardioplegia in maintaining cellular homeostasis. This observation is significant as it suggests that reperfusion injury was more severe in tissues lacking cardioplegia protection. Although this passive *ex vivo* reperfusion model lacks hemodynamic flow, it has been validated as a reliable method for assessing early-phase myocardial injury and recovery, particularly under standardized reperfusion conditions. To enhance physiological relevance, the reperfusion medium was modified with appropriate concentrations of potassium and calcium ions, which are critical for myocardial cellular repolarization and calcium homeostasis during reoxygenation. Our methodology was adapted from previously validated protocols employed in rodent models to assess the cardioprotective effects of cardioplegic solutions under controlled IR conditions, with modifications to better reflect whole-animal physiology [15]. Consistent with the light microscopic findings, we also observed greater nuclear disruption in the control group, as indicated by significantly increased karyolytic nuclear scores during reperfusion. Among the cardioplegia-treated groups, the del Nido group exhibited the lowest median karyolysis scores, suggesting a trend toward improved nuclear preservation. Beyond these histological findings, our inclusion of TEM analysis provided a detailed view of cardioplegia-mediated ultrastructural preservation. Under TEM, the distinct mitochondrial morphology and endothelial continuity observed in the del Nido group align with previous ultrastructural studies underscoring the importance of mitochondrial integrity in myocardial recovery [22, 23]. Mitochondria are central regulators of reperfusion injury, and maintaining their cristae organization is critical for ATP synthesis and calcium homeostasis. The lower Flameng scores and higher crista density in the del Nido group thus reflect meaningful biochemical preservation, even in the absence of hemodynamic reperfusion. These ultrastructural observations reinforce the concept that cardioplegic protection extends beyond inflammation to encompass cellular energy stability and structural resilience. This histopathological indicator of irreversible myocyte injury further supports the hypothesis that cardioplegia solutions help maintain cellular integrity under IR conditions. While these nuclear changes were less pronounced in both cardioplegia-treated groups, del Nido demonstrated slightly lower scores than St. Thomas II, indicating better preservation of nuclear morphology, though not reaching statistical significance. Furthermore, the ultrastructural evaluation by TEM revealed clear distinctions between the groups.

Electron microscopy revealed that the control myocardium exhibited extensive mitochondrial swelling, disrupted cristae, and thickened capillary basement membranes, indicative of advanced IR injury. St. Thomas II cardioplegia offered partial preservation; however, focal cristae rarefaction and sarcomeric disorganization remained evident. In contrast, del Nido cardioplegia maintained dense, well-organized mitochondrial cristae, thin endothelial basement membranes, and continuous Z-line alignment, demonstrating superior subcellular protection. Quantitative analysis confirmed that Flameng scores and basement membrane thickness were significantly lower, while crista density and mitochondrial form factor were significantly higher in the del Nido group compared to both the control and St. Thomas II groups (*P* < 0.05). These findings reinforce the protective effects of cardioplegia beyond inflammatory modulation.

Although 90 min of global ischemia typically induces pronounced histopathological injury in rodent myocardium, overt necrotic morphology on routine H&E staining may lag behind ultrastructural damage in non-perfused experimental settings. The passive, non-oxygenated immersion model utilized in our study does not involve active coronary flow, thereby limiting ionic washout and delaying cytoplasmic coagulation, membrane disruption, and striation loss typically observed in blood-based ischemia. Consistent with this, previous studies have demonstrated that early ischemic injury is more readily detectable by TEM than by H&E staining in diffusion-limited models. Our study’s TEM results clearly demonstrated mitochondrial swelling, crista disruption, endothelial injury, and sarcomeric disorganization, whereas H&E sections appeared relatively preserved, reflecting the known dissociation between ultrastructural injury and overt histological necrosis during the early ischemic period. These ultrastructural findings align with several previous experimental and clinical investigations. Similar electron microscopy studies in both experimental and clinical settings have shown that cardioplegic composition significantly influences mitochondrial and sarcomeric integrity. Jung et al. [15] conducted serial TEM evaluations in human myocardial samples infused with del Nido solution, reporting well-preserved cristae structure and reduced matrix swelling compared to traditional blood cardioplegia, which is consistent with our findings. Zakharova et al. [24] observed that modified St. Thomas cardioplegia provided partial mitochondrial protection but resulted in residual cristae disruption, paralleling the intermediate ultrastructural scores observed in our St. Thomas II group. Moreover, Lira et al. [25] published a study protocol outlining a planned comparison of del Nido and St. Thomas cardioplegia, rather than outcome data. These studies collectively support the hypothesis that del Nido confers superior subcellular preservation through enhanced ionic balance and free-radical scavenging. Our TEM results closely align with these observations, further validating del Nido’s ultrastructural advantage under controlled reperfusion conditions. However, at the light microscopic level, there were no statistically significant differences between the St. Thomas II and del Nido groups regarding mast cell counts, macrophage infiltration, or leukocytic inflammation scores.

Similar light microscopic histopathological studies have reported that the effects of del Nido and St. Thomas II cardioplegia solutions are largely comparable [26–28], a finding supported by recent comparative analyses by Çayır et al. [27]. For instance, in a study by Sen et al. [26], del Nido provided operative convenience without significantly affecting clinical outcomes. A retrospective analysis by Yamashita et al. [29] found that cross-clamp and CPB times were significantly shorter in patients who received del Nido, although markers of myocardial injury were similar. Likewise, a meta-analysis by Awad et al. [8], encompassing seven studies, reported that while del Nido reduced operative time, it did not differ from St. Thomas II in terms of mortality, arrhythmia, or inotrope requirements. These findings align with our study’s results and suggest that the histopathological advantages of del Nido may not be apparent in short-term reperfusion models.

Our results are largely consistent with these previous findings. From a histopathological perspective, the inflammatory response following reperfusion was more limited in both cardioplegia groups compared to controls, but no definitive superiority of del Nido was demonstrated. This may be attributable to the rat model’s resistance to short-term ischemia, the limited 30-min reperfusion duration, and the focus on the early phase of the systemic inflammatory response.

One of the most significant clinical advantages of del Nido is its ease of use and effectiveness for up to 90 min with a single dose [9, 30]. This facilitates uninterrupted surgical workflows, making it particularly beneficial for lengthy or minimally invasive procedures [20].

In terms of macrophage infiltration, a significant increase was observed in the control group, interpreted as evidence of an activated inflammatory cascade in tissue post-reperfusion. This finding indicates that early inflammatory responses to reperfusion injury are more pronounced in the absence of cardioplegia. Although both cardioplegia groups suppressed this macrophage response, the difference was not statistically significant. Similarly, our analysis of leukocytic infiltration showed no significant differences among the three groups, suggesting that cardioplegia administration may not substantially alter leukocyte recruitment during early reperfusion under the current model conditions. It is possible that longer reperfusion durations or the inclusion of biochemical markers could yield different outcomes. While light microscopic parameters such as mast cell density and nuclear preservation scores provided insight into tissue-level responses, a more robust characterization of the inflammatory cascade would require additional immunohistochemical markers. Markers such as CD68 for macrophages, myeloperoxidase (MPO) for neutrophils, or proinflammatory cytokines (e.g., IL-6, TNF-α) could offer deeper insights into the cardioprotective mechanisms of these solutions. However, our study design prioritized histological evaluation using standard staining techniques due to resource and methodological constraints. Future studies should incorporate these markers to delineate more precisely whether del Nido or St. Thomas II exerts superior anti-inflammatory effects.

The arrest mechanism induced by the high potassium concentration in St. Thomas II remains a reliable and effective classical method [7, 8, 11]. In contrast, del Nido is distinguished by its low viscosity and potential for better preservation of intracellular ionic homeostasis [8–10]. However, our study did not demonstrate a definitive histopathological advantage of these features. While the control group did not exhibit severely disrupted nuclear morphology or prominent inflammatory infiltration, which might be expected in an untreated IR model, this finding likely reflects the limitations of the current short-term reperfusion protocol. The absence of overt tissue destruction in the control group may suggest that a 30-min reperfusion period is insufficient to fully capture the extent of IR-induced histological damage. This methodological constraint reduces the model’s discriminatory power and should be considered when interpreting comparative histopathological outcomes. Additionally, the omission of confidence intervals for non-parametric effect sizes represents an inherent methodological limitation, and the conservative nature of the Bonferroni correction should be considered when interpreting the findings. Future studies incorporating extended reperfusion durations and molecular-level assays may provide a more realistic modeling of clinical IR injury. The use of TEM also enabled precise identification of mitochondrial, sarcomeric, and endothelial alterations that would have been missed under LM, thereby strengthening the histopathological interpretation.

In conclusion, both cardioplegia solutions were effective in reducing myocardial inflammation. Although del Nido attracted attention due to its ease of administration, targeted pharmacological effects, and single-dose advantage, it did not demonstrate significant histopathological superiority over St. Thomas II. These findings are consistent with existing clinical and experimental data. Nonetheless, future experimental studies incorporating longer reperfusion periods and molecular or immunohistochemical analyses are warranted.

### Limitations of the study

This study provides a comparative assessment of the histopathological effects of St. Thomas II and del Nido cardioplegia solutions on myocardial ischemia-reperfusion injury in a rat model. However, several limitations must be considered when interpreting the findings.

#### Model-related limitations

First, although the sample size of eight rats per group was adequate to maintain a certain level of statistical power, it may not have sufficiently captured biological variability. Larger sample sizes are necessary, particularly for histopathological evaluations, to more clearly delineate differences between groups. Additionally, due to the small sample size and the ordinal nature of several endpoints, we did not apply multivariable mixed-effects models to simultaneously analyze group, phase (ischemia vs reperfusion), and their interaction. Instead, ischemia and reperfusion measurements were assessed using phase-specific non-parametric tests with paired within-group comparisons.

Second, the reperfusion period was limited to 30 min. While this duration is sufficient to observe acute inflammatory responses, it is inadequate for evaluating late-phase inflammation, fibrosis, or macrophage-mediated tissue remodeling. This study utilized a well-defined animal model of IR injury, incorporating both surgical induction and cardioplegic arrest. However, we acknowledge that this model primarily captures early-phase changes within a limited reperfusion window. Because it does not include coronary flow, oxygen delivery, or metabolic substrate supplementation, the findings reflect early passive post-ischemic structural changes and should not be directly generalized to clinically perfused situations such as CPB. Incorporating longer reperfusion durations, serial sampling at multiple time points, or advanced histopathological techniques (such as immunohistochemistry) could provide a more comprehensive understanding of the evolving injury and repair mechanisms. Therefore, while our model effectively simulates acute myocardial injury, future studies employing more elaborate experimental timelines may better reflect the complex dynamics of reperfusion injury. Experimental protocols with extended reperfusion phases are required to improve clinical relevance.

Lastly, although the rat heart is a widely used model in experimental research, it differs from human myocardium in metabolic rate, ion channel profiles, and regenerative capacity. Consequently, the direct generalization of these findings to clinical practice should be approached with caution, and future studies should incorporate large animal models.

#### Marker-related and analytical limitations

Third, this study utilized only classical histological stains such as toluidine blue and hematoxylin-eosin. The absence of immunohistochemical techniques limits the ability to quantitatively distinguish specific cell types—such as macrophages identified by CD68, neutrophils by MPO, or T lymphocytes by CD3—and to characterize the inflammatory response at a cellular level. While intramyocardial temperature was not measured with an implantable sensor, myocardial surface temperature was consistently maintained at 30—32 ^∘^C by placing sterile ice packs near the heart without direct contact. A calibrated surface thermometer was used throughout the ischemia period to ensure stable hypothermic conditions.

Another limitation is the lack of biochemical markers of myocardial damage (e.g., troponin I or T, CK-MB, LDH). Correlating histological findings with such serum indicators would strengthen the relationship between structural and functional injury.

### Overall interpretation

Despite these limitations, this study provides original data comparing the histopathological effects of two different cardioplegia solutions under controlled IR conditions. It demonstrates that both solutions are comparably effective in managing early inflammatory responses, with del Nido showing a trend toward better stabilization of cellular inflammation.

## Conclusion

In this experimental study, we compared the myocardial protective effects of St. Thomas II and del Nido cardioplegia in a rat model of IR. Light microscopic evaluation revealed that both solutions effectively reduced mast cell activation, leukocyte infiltration, and nuclear damage compared to the control group. Although statistical differences were not significant, del Nido cardioplegia demonstrated slightly better preservation of cellular morphology and a more stable inflammatory profile than St. Thomas II.

TEM further highlighted this distinction, showing that del Nido provided superior mitochondrial and endothelial integrity, while St. Thomas II offered moderate but incomplete ultrastructural preservation. Overall, both solutions were effective, but del Nido appeared to confer more comprehensive protection at both histological and ultrastructural levels. Further studies with extended reperfusion periods and biochemical validation are necessary to confirm these findings.

## Supplemental data

**Key points**

**a. What is known about the topic?**

- Myocardial ischemia–reperfusion injury is a significant contributor to perioperative morbidity in cardiac surgery.
- Cardioplegia solutions, such as St. Thomas II and del Nido, are commonly employed to mitigate ischemic damage; however, comparative histological and ultrastructural evidence from experimental studies remains scarce.
- While del Nido cardioplegia presents pharmacological advantages due to its components, lidocaine and mannitol, St. Thomas II has traditionally been regarded as a reliable standard for myocardial protection.

**b. What does this study add?**

- This study offers the first comprehensive comparison of St. Thomas II and del Nido cardioplegia using integrated LM and transmission electron microscopy in a controlled rat ischemia–reperfusion model.
- Both cardioplegia solutions effectively preserved myocardial histological architecture, stabilized mast cell counts, and reduced inflammatory cell infiltration compared to untreated controls.
- Transmission electron microscopy, the predefined primary endpoint of this study, revealed statistically significant ultrastructural superiority of del Nido cardioplegia over St. Thomas II—characterized by lower Flameng scores, higher crista density, improved Z-line continuity, and thinner basement membranes.
- These findings suggest that, although light microscopic parameters appear comparable between the two solutions, del Nido cardioplegia offers enhanced mitochondrial and microvascular preservation at the ultrastructural level.

## Data Availability

The datasets generated and/or analyzed during the current study are available from the corresponding author upon reasonable request.
